# Exosomes as a predictor tool of acquired resistance to melanoma treatment

**DOI:** 10.1186/1753-6561-8-S4-P28

**Published:** 2014-10-01

**Authors:** Lucas Goedert, Richard Koya, Siwen Hu-Lieskovan, Antoni Ribas

**Affiliations:** 1 Federal University of Pelotas, Rio Grande do Sul, Brazil; 2 Universityof California Los Angeles, Los Angeles, CA, USA

## Background

Exosomes are small endosome-derived vesicles ranging from 30-120 nm in diameter secreted from cells through exocytosis and, in cancer context, are able to prepare metastasis niche and suppress host's immune system. Despite these pro-tumorigenic characteristics, exosomes can be useful for several Biotechnological applications such as biological anticancer vaccines, cancer diagnosis and prognosis. Due to its property of carrying parental cell's protein and active nucleic acids there is an increasing interest to use exosomes to track cancer metabolism alterations in different conditions, for example, during drug administration [1].

One of the most common types of cancer that develops drug resistance during drug treatment is Melanoma. The majority of drugs developed against melanoma targets the oncogenic mutations in BRAF (BRAF V600E) that are present in nearly 60% of cutaneous melanoma tumors. Resistance to BRAFV600E inhibitors are classified in two main groups, Intrinsic Resistance and Acquired Resistance, the second is developed after treatment starts that happens mainly by MAPK reactivation and RTK upregulation [2].

Various methods are being studied to overcome drug resistance in melanoma, but there is a need to detect when the resistant mechanism is arising to orientate which approach to choose. This early detection has demonstrated to be a difficult task especially during advanced stages where there are several metastatic niches.

With this aim, our research project was designed to create a method to follow tumor's status through melanoma-derived exosomes. As most types of melanoma overexpress MART-1 we checked its expression, as well as PDGFR-B and others proteins, in PLX4032 (V600E mutated BRAF inhibitor) resistant and sensitive melanoma cell lines to start to understand their potential to be exosomes-associated proteins in the development of acquired resistance detection method[2].

## Methodology

Cells were cultured in exosome depleted media by FBS ultracentrifugation during 70 min at 100.000xg. Sensitive (M229, M238, M249) and resistant (M229AR, M238AR, M249AR) melanoma cell lines to PLX 4032 (vemurafenib) were cultured until 75% of confluence and the supernatant was processed to obtain the exosomes following 1500x g - 10 min, 17000x g - 15min and 160.000x g - 1 hour.

## Results

To confirm this protocol it was done a Transmission Electron Microscopy (TEM), followed by Flow Cytometry against CD63, an exosome molecular marker. TEM showed an uniform size for exosomes ranging from 60- 90 nm in diameter and Anti-CD63 Flow Cytometry showed a positive population of 99.1%. Western Blots were done with the cells lines cited previously demonstrating that the exosome's protein content of MART-1, PDGFR-B, FGFR3 and others, corresponds to the parental cell.

## Conclusion

Preliminary results show that exosomes can be useful tools to predict cell's metabolism. Here we present that protein differential expression between sensitive and resistant cell lines can be detected by exosome profile such as PDGFR-B increased expression in resistant lines.
